# On the evolutionary relationship between chondrocytes and osteoblasts

**DOI:** 10.3389/fgene.2015.00297

**Published:** 2015-09-23

**Authors:** Patsy Gómez-Picos, B. Frank Eames

**Affiliations:** Department of Anatomy and Cell Biology, University of Saskatchewan, Saskatoon, SKCanada

**Keywords:** EvoDevo, comparative transcriptomics, Sox9, Runx2, bone, cartilage, GRN

## Abstract

Vertebrates are the only animals that produce bone, but the molecular genetic basis for this evolutionary novelty remains obscure. Here, we synthesize information from traditional evolutionary and modern molecular genetic studies in order to generate a working hypothesis on the evolution of the gene regulatory network (GRN) underlying bone formation. Since transcription factors are often core components of GRNs (i.e., kernels), we focus our analyses on Sox9 and Runx2. Our argument centers on three skeletal tissues that comprise the majority of the vertebrate skeleton: immature cartilage, mature cartilage, and bone. Immature cartilage is produced during early stages of cartilage differentiation and can persist into adulthood, whereas mature cartilage undergoes additional stages of differentiation, including hypertrophy and mineralization. Functionally, histologically, and embryologically, these three skeletal tissues are very similar, yet unique, suggesting that one might have evolved from another. Traditional studies of the fossil record, comparative anatomy and embryology demonstrate clearly that immature cartilage evolved before mature cartilage or bone. Modern molecular approaches show that the GRNs regulating differentiation of these three skeletal cell fates are similar, yet unique, just like the functional and histological features of the tissues themselves. Intriguingly, the Sox9 GRN driving cartilage formation appears to be dominant to the Runx2 GRN of bone. Emphasizing an embryological and evolutionary transcriptomic view, we hypothesize that the Runx2 GRN underlying bone formation was co-opted from mature cartilage. We discuss how modern molecular genetic experiments, such as comparative transcriptomics, can test this hypothesis directly, meanwhile permitting levels of constraint and adaptation to be evaluated quantitatively. Therefore, comparative transcriptomics may revolutionize understanding of not only the clade-specific evolution of skeletal cells, but also the generation of evolutionary novelties, providing a modern paradigm for the evolutionary process.

## Introduction: Cartilage and Bone might Share an Evolutionary History

Most of evolutionary theory has focussed on studies of morphological change (morphogenesis) among taxa, but the formation of tissue types (histogenesis) also can evolve in clade-specific manners. Therefore, we focus our attentions on a relatively understudied subject of evolutionary research: the evolution of histogenesis. A classic problem in evolutionary theory is to explain novelties, or traits with no clear ancestral antecedent (Shubin, 2002; Moczek, 2008; Wagner and Lynch, 2010). For example, vertebrates are the only animals that produce bone, but so far, the molecular genetic basis for this evolutionary novelty remains obscure. Here, we synthesize information from traditional evolutionary and modern molecular studies in order to generate a working hypothesis on the evolution of the genetic system underlying bone formation. Many studies argue that bone evolved from dentine (Kawasaki et al., 2004; Wagner and Aspenberg, 2011). However, using molecular genetic and embryological arguments that favor gradualism over saltationism (Gould, 2002), we hypothesize that bone (and perhaps all mineralizing tissues, such as dentine) appeared during evolution by co-opting a gene regulatory network (GRN) that was under prior natural selection to mineralize cartilage. In order to present an argument for skeletal tissue development and evolution over the past 500 million years, we make some generalizations that may trouble some readers, of whom we ask their indulgence, hoping that such generalizations help to reveal broader trends during the evolution of skeletal tissues.

**FIGURE 1:**
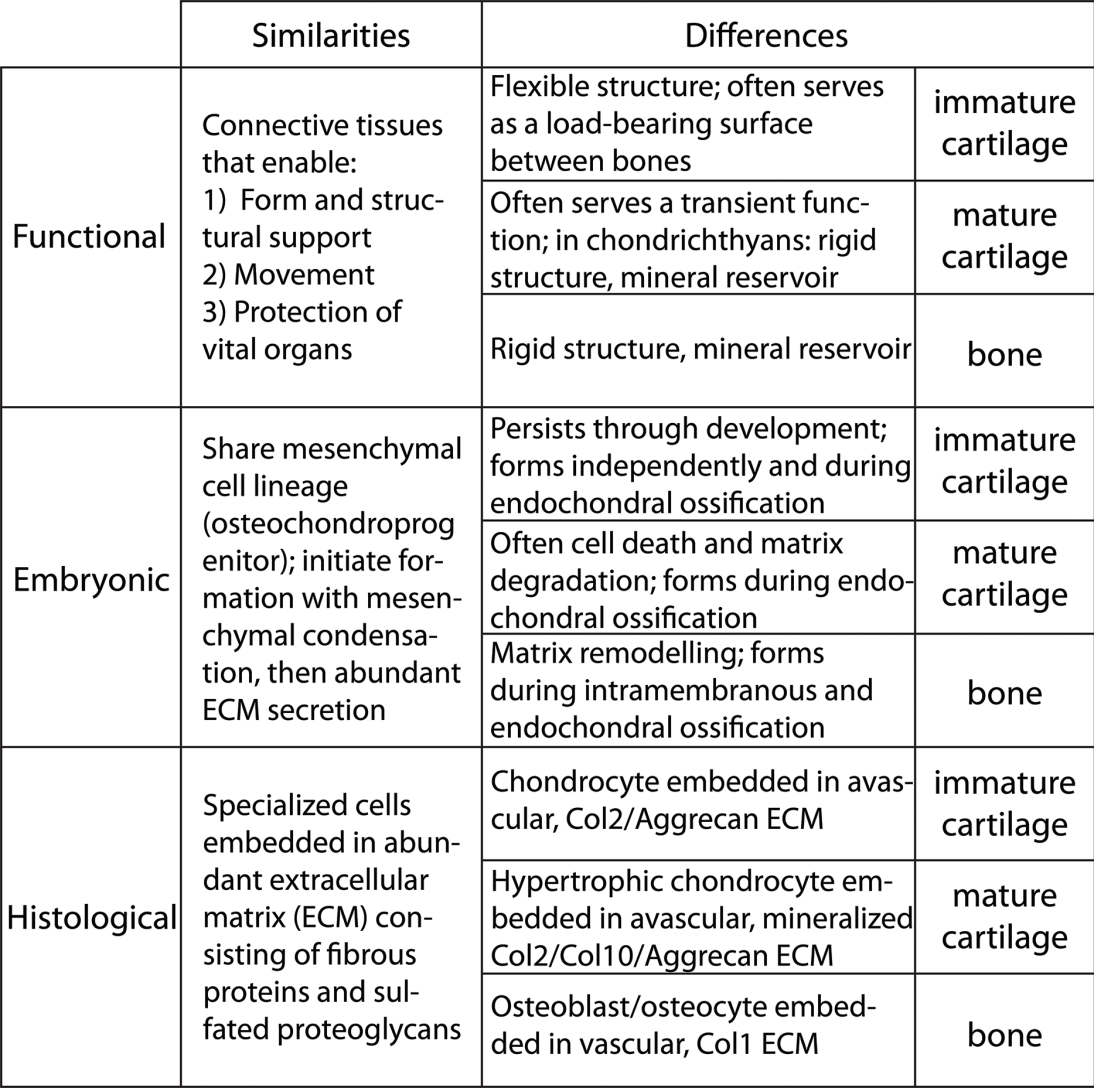
**Similarities and differences among immature cartilage, mature cartilage, and bone suggest that these three skeletal tissues share an evolutionary history**.

An introductory look at the similarities and differences among cartilage and bone suggests that the underlying GRNs may be related. Cartilage and bone are specialized connective tissues that provide form and structural support to the body, protect vital organs, and play a crucial role in locomotion through muscle attachments (Gray and Williams, 1989). Despite these similarities, they also have distinct functions (**Figure 1**). Cartilage typically offers a flexible structure to support soft tissues and also to serve as a load-bearing surface between bones. On the other hand, bone is a hard, rigid structure that protects vital organs and acts as a storage site for minerals, such as calcium and phosphorus (Smith and Hall, 1990; Volkmann and Baluska, 2006). Also unlike cartilage, which has almost no capacity for regeneration, bone is a highly dynamic structure that undergoes constant remodeling, preserving bone strength and regulating calcium homeostasis (Datta et al., 2008). Perhaps related to regenerative capacity, these tissues differ in vascularity. Bone is highly vascularized, but cartilage typically is avascular. However, important exceptions to cartilage vascularization occur. Mature cartilage in tetrapods often is invaded by vasculature as it degrades, creating the marrow cavity (Johnson, 1980; Roach, 1997; Stricker et al., 2002; Ortega et al., 2004; Moriishi et al., 2005), and even immature cartilage is highly vascularized near articulating surfaces in some avian and mammalian species (Ytrehus et al., 2004; Blumer et al., 2005). When cartilage extracellular matrix (ECM) undergoes mineralization, its functions change. In some vertebrates, such as sharks, mineralized cartilage can serve as the major rigid structural support for the body, meanwhile providing a mineral reservoir (Daniel, 1934; Kemp and Westrin, 1979; Eames et al., 2007). In most extant vertebrates, however, mineralized cartilage mainly serves as a scaffold during endochondral ossification, outlined below.

During embryonic development, cartilage and bone formation share many features (**Figure 1**). Both cartilage and bone are differentiated from common mesenchymal (osteochondral) progenitor cells (Fang and Hall, 1997; Day et al., 2005; Hill et al., 2005). Both cartilage and bone initiate overt differentiation by aggregating mesenchymal cells into condensations, which can go on directly to secrete cartilage- or bone-specific matrix (Hall and Miyake, 1995, 2000; Kronenberg, 2003; Day et al., 2005). However, a unique feature of bone formation is that, in addition to differentiating directly from an osteogenic condensation (intramembranous ossification), bone also forms on a pre-existing cartilage template (endochondral ossification). Endochondral ossification actually involves the formation of the three skeletal tissues that comprise the majority of the extant vertebrate skeleton: immature cartilage, mature cartilage and bone (Eames et al., 2003, 2004; Eames and Helms, 2004). Some cartilage remains throughout development at the growth plates and throughout life at articular surfaces (we term this immature cartilage). Most of the cartilage produced during endochondral ossification, however, undergoes a series of changes, termed maturation (thus the terms immature vs. mature cartilage). In most vertebrates, cartilage maturation involves cell hypertrophy, matrix mineralization, cell death, and matrix degradation (Leboy et al., 1988; Hatori et al., 1995; Takeda et al., 2001; Miura et al., 2008). Although exceptions exist (Thorogood, 1988; Hirasawa and Kuratani, 2015), endochondral ossification typically gives rise to the bones of the endoskeleton, such as the chondrocranium or limb skeleton, whereas intramembranous ossification produces the exoskeleton, such as lateral plates in teleosts or the calvarium (Smith and Hall, 1990).

Histologically, immature cartilage, mature cartilage, and bone are very similar, yet each also has some unique features (**Figure 1**). All three skeletal tissues are comprised of cells embedded in an ECM that is rich in collagens and proteoglycans (Hardingham, 1981; Eames et al., 2003, 2004; Eames and Helms, 2004; Gentili and Cancedda, 2009). Immature cartilage is formed by chondrocytes that deposit a network of loose collagen fibers and a rich substance of proteoglycans, whereas chondrocytes of mature cartilage alter the immature cartilage ECM by decreasing its proteoglycan sulfation and mineralizing it (Lohmander and Hjerpe, 1975; Buckwalter et al., 1987; Bayliss et al., 1999). The requirement of proteoglycan degradation for mature cartilage ECM mineralization is debated (Hirschman and Dziewiatkowski, 1966; Granda and Posner, 1971; Poole et al., 1982; Campo and Romano, 1986). Bone is formed by osteoblasts that produce an ECM of tightly wound and highly cross-linked collagen fibers, and bone ECM has lower levels of proteoglycans than cartilage (Gentili and Cancedda, 2009). As a result of these collagen and proteoglycan concentrations, these three skeletal tissues have overlapping and unique histological staining patterns. High concentrations of sulfated proteoglycans cause immature cartilage to stain with Alcian blue and Safranin O (by comparison, mature cartilage and bone bind these dyes with decreasing intensity, respectively). The tightly wound collagen fibers of bone stain with Direct red and Aniline blue (by comparison, loose collagen fibers of cartilage matrix bind these dyes with lower intensity; Villanueva et al., 1983; Hall, 1986; Eames and Helms, 2004; Eames et al., 2004, 2007). Alizarin red can stain mineralized tissues of mature cartilage and bone (Hogg, 1982; Kirsch et al., 1997; Eames and Helms, 2004; Eames et al., 2007).

Immature cartilage, mature cartilage, and bone have overlapping, but distinct, gene and protein expression profiles (**Figure 1**). All these skeletal tissues express Collagen 11 and the proteoglycans Biglycan and Decorin (Li et al., 1998; Knudson and Knudson, 2001; Rees et al., 2001; Roughley, 2006). Immature cartilage expresses high levels of Collagens 2 and 9, as well as the proteoglycans Aggrecan, Fibromodulin, and Epiphycan, which distribute growth factors and provide swelling pressure due to water attraction (Yanagishita, 1993; Lefebvre et al., 1997; Lefebvre and de Crombrugghe, 1998; Watanabe et al., 1998; Liu et al., 2000). Mature cartilage has reduced expression of these same collagens and proteoglycans, while also expressing high levels of Collagen 10 (Orth et al., 1996; Eames et al., 2004; Talwar et al., 2006). In contrast to both types of cartilage, bone expresses high levels of Collagen 1 (Yasui et al., 1984; Kream et al., 1995). Interestingly (and central to the argument of this review), both mature cartilage and bone share expression of genes not expressed in immature cartilage, including *Sp7* (formerly called *Osterix)*, *Matrix metallopeptidase 13* and *Indian hedgehog* (Vortkamp et al., 1996; Inada et al., 1999; Neuhold et al., 2001; Zaragoza et al., 2006; Abzhanov et al., 2007; Mak et al., 2008; Huycke et al., 2012; Nishimura et al., 2012; Weng and Su, 2013). In fact, very few genes expressed in bone are not expressed in mature cartilage, and this list of genes decreases further when comparisons among mature cartilage and bone are carried out in actinopterygians (Eames et al., 2012). Multiple genes associated with matrix mineralization are expressed in both mature cartilage and bone, such as *Alkaline phosphatase, liver/bone/kidney* (*Alpl*, formerly called *Tissue-nonspecific alkaline phosphatase*), *Secreted phosphoprotein 1* (*Spp1*, formerly called *Osteopontin* or *Bone sialoprotein*), *Secreted protein, acidic, cysteine-rich* (*Sparc*, formerly called *Osteonectin*), and *Bone gamma-carboxyglutamate protein* (*Bglap*, formerly called *Osteocalcin*; Termine et al., 1981; Pacifici et al., 1990; Chen et al., 1991; Bonucci et al., 1992; McKee et al., 1992; Mundlos et al., 1992; Nakase et al., 1994; Roach, 1999; Sasaki et al., 2000).

Currently, the evolutionary relationship among skeletal tissues is unclear, but the similarities highlighted above suggest that immature cartilage, mature cartilage, and bone share an evolutionary history. From a molecular genetic perspective, these observations lead to the hypothesis that the GRNs governing the formation of these three skeletal tissues (in particular, the differentiation of three skeletal cell types) also share an evolutionary history. Indeed, the many varieties of skeletal tissues intermediate between cartilage and bone observed in extant and fossil vertebrates may owe their existence to this shared history (Benjamin, 1990; Benjamin and Ralphs, 1991; Benjamin et al., 1992; Mizoguchi et al., 1997; Hall, 2005; Witten et al., 2010). In this review, we explore this hypothesis using traditional evolutionary and modern molecular genetic studies. We are not focussing on the exact anatomical location of a tissue, given that once the GRN regulating formation of that skeletal tissue is established in the genome, any cell in the body can co-opt its expression. Traditional studies have provided insight into the evolutionary relationship among skeletal tissues, since they demonstrate that immature cartilage originated first during phylogeny (Mallatt and Chen, 2003; Rychel et al., 2006). Interestingly, modern molecular genetic studies reveal that two GRNs dictate the formation of these three skeletal tissues (Bi et al., 1999; Inada et al., 1999; Eames et al., 2004; Hattori et al., 2010; Leung et al., 2011), and also that the GRN underlying cartilage formation is dominant to that of bone (Eames et al., 2004; Zhou et al., 2006). We expand upon this finding using an argument based on the relative parsimony of gradualism versus saltationism to hypothesize that bone evolved from a cartilage maturation program. In closing, we discuss how comparative transcriptomics will enhance dramatically our ability to test hypotheses on the evolution of the GRNs underlying cartilage and bone formation.

## GRN Underlying Immature Cartilage Formation Evolved First

**FIGURE 2:**
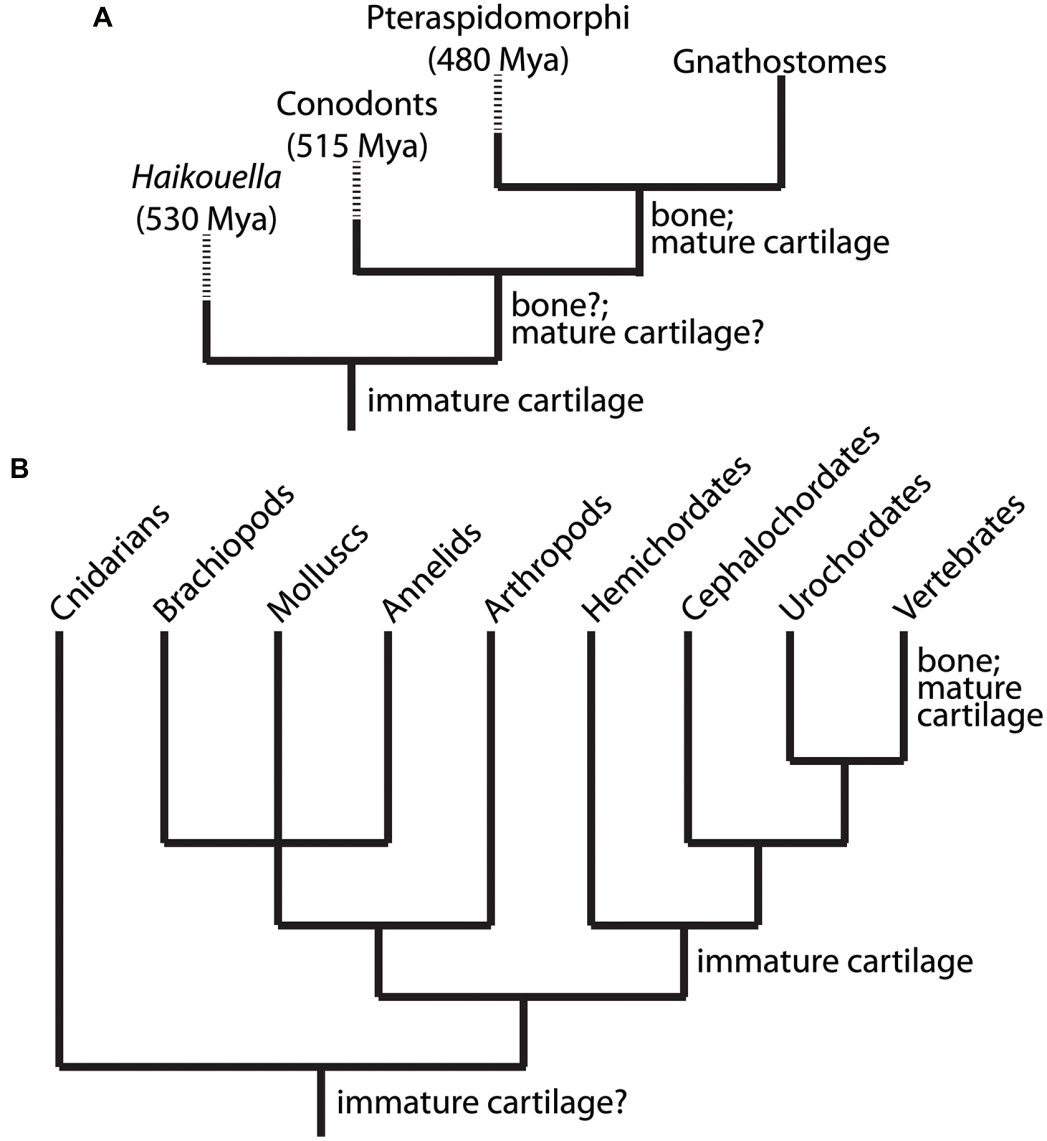
**Clues to the evolutionary relationship between the chondrocyte and osteoblast emerge from analyses of the fossil record and comparative anatomy. (A)** Appearance of immature cartilage, mature cartilage, and bone from available fossil record. These data indicate clearly that immature cartilage appeared first, then mature cartilage and bone. By extension, the chondrocyte preceded the osteoblast during evolution. **(B)** Extant taxa with at least one species containing cartilage or cartilage-like tissues, which are non-mineralized outside of vertebrates. These data suggest that a GRN driving differentiation of an immature chondrocyte evolved first, and then became established in the genome of chordates (along with the notochord, a cartilage-like tissue). Subsequently, this GRN was modified by another GRN that drove differentiation of a mature chondrocyte (and osteoblast) within vertebrates. Branch lengths in trees are arbitrary; dashed lines indicate extinct taxa.

Traditional studies, such as the fossil record, comparative anatomy, and embryology, demonstrate that the first skeletal tissue to evolve was immature cartilage (**Figure 2**). The fossil record reveals a great diversity of mineralized tissues about 500 million years ago (Mya; Janvier, 1996, 2015; Donoghue and Sansom, 2002; Donoghue et al., 2006), suggesting that GRNs of skeletal histogenesis were undergoing an adaptive radiation. So which skeletal tissue appeared first in the fossil record? This question is complicated by the facts that currently discovered fossils may represent a biased fraction of ancestral tissues, and that non-mineralized, lightly mineralized, or transiently mineralized tissues likely are not preserved well in the fossil record. Despite these limitations, however, the oldest skeletal tissue in the fossil record is unmineralized cartilage in the chordate fossil *Haikouella* from 530 Mya (**Figure 2A**; Mallatt and Chen, 2003). Many specimens preserving soft tissues of this incredibly important fossil have been found, but they appear to be represented only in a small region of the Yunnan province in China (Chen et al., 1999), reflecting potential bias in the fossil record.

Bone and mature cartilage appeared much later than immature cartilage in the fossil record (**Figure 2A**). Conodonts, a group of agnathans (jawless vertebrate fish), are the earliest (~515 Mya) known fossils with a mineralized skeleton, characterized by pharyngeal tooth-like elements comprised of tissues that were bone-like, enamel-like, and mineralized cartilage-like (Sansom et al., 1992). However, subsequent analyses of conodont fossils refuted the conclusion that bone or mineralized cartilage was present in these primitive jawless fish, instead attributing the first appearance of bone in the fossil record to the exoskeleton of pteraspidormorphi (~480 Mya), a group of armored agnathans (Janvier, 1996; Donoghue, 1998; Donoghue et al., 2006). Interestingly, some pteraspidomorph species (e.g., eriptychiids and arandaspids) and other, primitive fossil fish show traces of both mineralized cartilage and bone in their endoskeleton (Janvier, 1996, 1997; Zhang et al., 2009). Also, fossils of the ancestral vertebrate *Palaeospondylus gunni* (~385 Mya) reveal an entire adult skeleton comprised of hypertrophic, mineralized cartilage, while bone is completely absent (Johanson et al., 2010). Despite these findings, the current fossil record generally suggests that bone preceded mineralized cartilage (Smith and Hall, 1990; Janvier, 1997; Donoghue et al., 2006), although the molecular genetic and embryological arguments of this review call into question the accuracy of this conclusion. What is clear from the fossil record is that unmineralized cartilage was the first skeletal tissue to appear leading to the evolution of vertebrates (Northcutt and Gans, 1983; Smith and Hall, 1990).

Comparative anatomy also supports the notion that immature cartilage was the first skeletal tissue to evolve, because immature cartilage is distributed in a broader range of taxonomic lineages than mature cartilage or bone (**Figure 2B**). Immature cartilage appears in both vertebrate and non-vertebrate species, whereas mature cartilage and bone are shared, derived traits of vertebrates only (Cole and Hall, 2004, 2009; Rychel et al., 2006). In a seminal study by Cole and Hall (2004), cartilage was demonstrated in a variety of taxonomically distinct invertebrates, such as polychaetes, arthropods, and molluscs. Reflecting the different evolutionary histories of immature and mature cartilage, cartilage in any invertebrate lineage, and also in extant agnathans, is unmineralized (Cole and Hall, 2004; Hall, 2005). The finding that lamprey cartilage can mineralize *in vitro* suggests that early agnathans may have possessed mineralized cartilage and these mineralization programs were repressed in cyclostomes (Langille and Hall, 1993).

The taxonomic distribution of cartilage suggests that the ancestor of vertebrates, cephalochordates, and hemichordates had an ability to make immature cartilage (**Figure 2B**). In fact, the deuterostome ancestor was proposed to be a benthic worm with cartilaginous gill slits (Rychel et al., 2006). Homology between invertebrate and vertebrate cartilages is supported by biochemical and histological analyses, which demonstrate high amounts of fibrous proteins and mucopolysaccharides (Cole and Hall, 2004; Cole, 2011). In fact, recent studies have shown that the cirri in amphioxus share many histological and molecular features with vertebrate immature cartilage (Kaneto and Wada, 2011; Jandzik et al., 2015). However, homology between deuterostome and protostome cartilage is still uncertain and must be confirmed by modern molecular analyses, including examination of gene expression patterns, GRN architectures, and GRN regulation. The ECM of hemichordate skeletal tissues may show features of both cartilage and bone (Cole and Hall, 2004), supporting the notion that these two tissues share an evolutionary history. Mineralized cartilage and bone, however, are only found in extant gnathostomes (**Figure 2**). These comparative anatomy analyses suggest that immature cartilage evolved before mature cartilage and bone.

Final support for the idea that cartilage arose earlier in evolution than mature cartilage and bone comes from comparative embryology. While the Biogenetic Law of Ernst Haeckel definitely has its theoretical problems (Haeckel, 1866), a general correlation (recapitulation) between the timing of events during ontogeny with events during phylogeny is undeniable. Indeed, many early evolutionary biologists assumed this to be true (Gould, 2002). In this context, it is interesting to note that immature cartilage is the first skeletal tissue to undergo histogenesis during embryonic development, while cartilage maturation and bone formation are later events. The relative timing of cartilage maturation to bone formation, on the other hand, appears to vary among vertebrate taxa (Mori-Akiyama et al., 2003; Eames et al., 2004, 2012; Moriishi et al., 2005). While such relationships between the timing of developmental events have been argued to reflect simply the increasing complexity of ontogeny during phylogeny (Wallace, 1997), we believe that this issue, which has been debated for 100s of years, remains unresolved.

To sum up traditional studies of the fossil record, comparative anatomy, and embryology, the ability to make immature cartilage predates the ability to make mature cartilage or bone during evolution. Therefore, from a molecular genetic perspective, the GRN governing chondrocyte differentiation clearly appeared prior to that of the osteoblast. However, traditional approaches are still unclear whether mature cartilage or bone appeared next during evolution. With hopes that modern molecular and embryological analyses can shed light into the evolutionary origins of the vertebrate skeleton, we next discuss how the GRNs underlying the formation of immature cartilage, mature cartilage, and bone are organized.

## Sox9 GRN is Dominant to the Runx2 GRN

**FIGURE 3:**
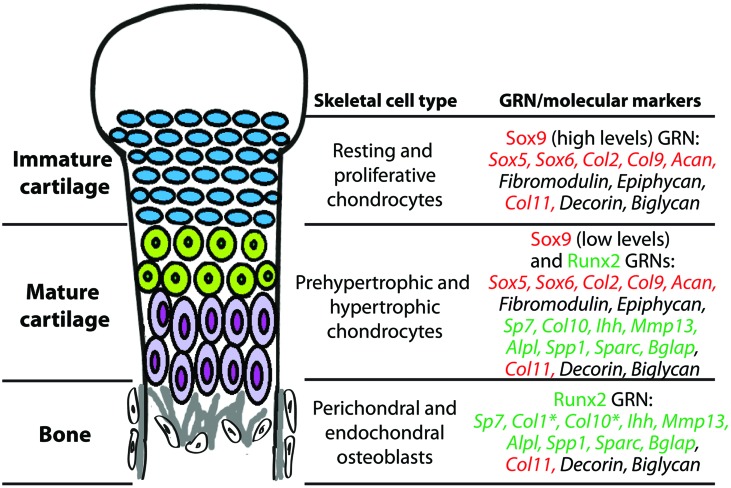
**During endochondral ossification, immature cartilage, mature cartilage, and bone differentiate under the control of Sox9 and Runx2 GRNs.** Chondrocytes of immature cartilage, termed resting and proliferative chondrocytes during endochondral ossification, express high levels of genes in the Sox9 GRN. Genes known to be under direct transcriptional control of Sox9 or Runx2 are highlighted in red or green text, respectively. Chondrocytes of mature cartilage, termed prehypertrophic and hypertrophic chondrocytes during endochondral ossification, express low levels of genes in the Sox9 GRN and also genes in the Runx2 GRN. Osteoblasts in perichondral and endochondral bone during endochondral ossification express genes in the Runx2 GRN. *Col1 is one of the only genes expressed in osteoblasts that is not expressed in mature chondrocytes; Col10 expression in osteoblasts is high only in some vertebrates. Col11, Decorin, and Biglycan are expressed in all three of these skeletal cell types. Similar gene expression patterns are seen in immature cartilage, mature cartilage, and bone developing in the articular surface (not shown).

Skeletal histogenesis is governed by complex sets of genes, largely controlled by central transcription factors that are responsible for determining cell fate decisions (Eames et al., 2003, 2004; Kronenberg, 2003; Karsenty et al., 2009). Molecular genetic experiments demonstrate that the transcription factors Sox9 and Runx2 are the “master regulatory genes” of skeletal histogenesis. Sox9 and Runx2 expression patterns during mesenchymal condensation predict whether osteochondroprogenitor cells differentiate into immature cartilage, mature cartilage, or bone (Eames and Helms, 2004; Eames et al., 2004). Loss of Sox9 function abrogated immature and mature cartilage formation (Bi et al., 1999; Mori-Akiyama et al., 2003), whereas Runx2 loss of function blocked mature cartilage and bone formation (Hoshi et al., 1999; Inada et al., 1999; Kim et al., 1999; Enomoto et al., 2000). In gain-of-function experiments, Sox9 mis-expression induced ectopic cartilage formation, whereas Runx2 mis-expression induced ectopic mature cartilage and bone formation (Eames et al., 2004). These and other experiments show clearly that a Sox9 GRN regulates immature cartilage formation, a Runx2 GRN drives bone formation, and a combination of Sox9 and Runx2 GRNs produce mature cartilage (**Figure 3**). We emphasize the relevance of these transcription factors to the evolution of GRNs underlying skeletal histogenesis, since conserved, core components of GRNs (i.e., kernels) are often transcription factors (Levine and Davidson, 2005; Davidson and Erwin, 2006).

Expression studies of skeletal tissues in a range of organisms suggest an ancestral interaction between Sox and Runx GRNs. *Runx2*, along with its related family members, *Runx1* and *3*, derive from gnathostome duplications of an ancestral *Runx*, while agnathan *Runx* genes may have undergone an independent duplication (Meulemans and Bronner-Fraser, 2007; Hecht et al., 2008; Cattell et al., 2011; Kaneto and Wada, 2011; Nah et al., 2014). *Sox9*, along with its related family members, *Sox8* and *10*, derive from duplications to the ancestral *SoxE*, while agnathan *SoxE* genes may have undergone an independent duplication (Meulemans and Bronner-Fraser, 2007; Ohtani et al., 2008; Yu et al., 2008; Cattell et al., 2011; Uy et al., 2012; Jandzik et al., 2015). *Runx* and *SoxE* orthologs are expressed in cartilage of amphioxus, lamprey, and hagfish, suggesting that the gene ancestral to Runx2 primitively functioned with the gene ancestral to Sox9 in early cartilage formation (Hecht et al., 2008; Wada, 2010; Kaneto and Wada, 2011). Notably, these animals do not have bone, and they do not mineralize their skeletons. Interestingly, the amphioxus cirral skeleton shows features of both cartilage and bone, suggesting that this ancient skeleton might have diverged to form cellular cartilage and bone of vertebrates (Kaneto and Wada, 2011). We argue that evaluating the interactions between Sox9 and Runx2 GRNs leads to a novel hypothesis for the evolution of bone.

Many studies in mammals and chick demonstrate that the Sox9 GRN is at least partially dominant to the Runx2 GRN. First, co-expression of Sox9 and Runx2 typically causes cartilage formation, not bone (Eames and Helms, 2004; Eames et al., 2004). Second, ectopic expression of Sox9 in Runx2-expressing cells of developing bone (achieved either normally during secondary cartilage formation or experimentally using Sox9 mis-expression) diverts the cells to make cartilage, whereas ectopic Runx2 expression in Sox9-expressing cells of developing cartilage does not divert them to make bone (Eames et al., 2004). Third, Sox9 expression needs to be down-regulated in order for the full Runx2-dependent cartilage maturation program to be expressed (Akiyama et al., 2002; Eames et al., 2004). Fourth, Sox9 over-expression can inhibit Runx2 expression (Eames et al., 2004). Finally, and most conclusively, Sox9 directly binds to Runx2, inhibits its transcriptional activity, and increases ubiquitin-mediated degradation of Runx2 (Zhou et al., 2006; Cheng and Genever, 2010).

Given evidence that the Sox9 GRN can dominate the Runx2 GRN, the formation of mature cartilage during endochondral ossification, which requires both Sox9 and Runx2, must be regulated exquisitely (**Figure 3**). During early stages, both Sox9 and Runx2 are co-expressed in mesenchymal condensations (Akiyama et al., 2002; Eames and Helms, 2004; Eames et al., 2004; Zhou et al., 2006), so Sox9 must exert a dominant inhibitory effect over Runx2 in order to produce immature cartilage. Later, Sox9 is down-regulated and Runx2 activity increases, triggering cartilage maturation (Eames et al., 2004; Yoshida et al., 2004; Hattori et al., 2010). In fact, Sox9 down-regulation is a crucial step for mature cartilage formation (Hattori et al., 2010). Despite this down-regulation, a role for Sox9 in very late stages of cartilage maturation also has been revealed (Ikegami et al., 2011; Dy et al., 2012). One study even suggests that Runx2 can inhibit Sox9 activity (Cheng and Genever, 2010), illustrating that complex feedback mechanisms are in place to achieve the appropriate relative levels of Sox9 and Runx2 activity. In summary, the preponderance of published literature on molecular genetics demonstrates that Sox9 has dominant effects over Runx2, and we extend this conclusion to generate a new hypothesis on the evolution of bone.

## Bone Evolved from Mature Cartilage

**FIGURE 4:**
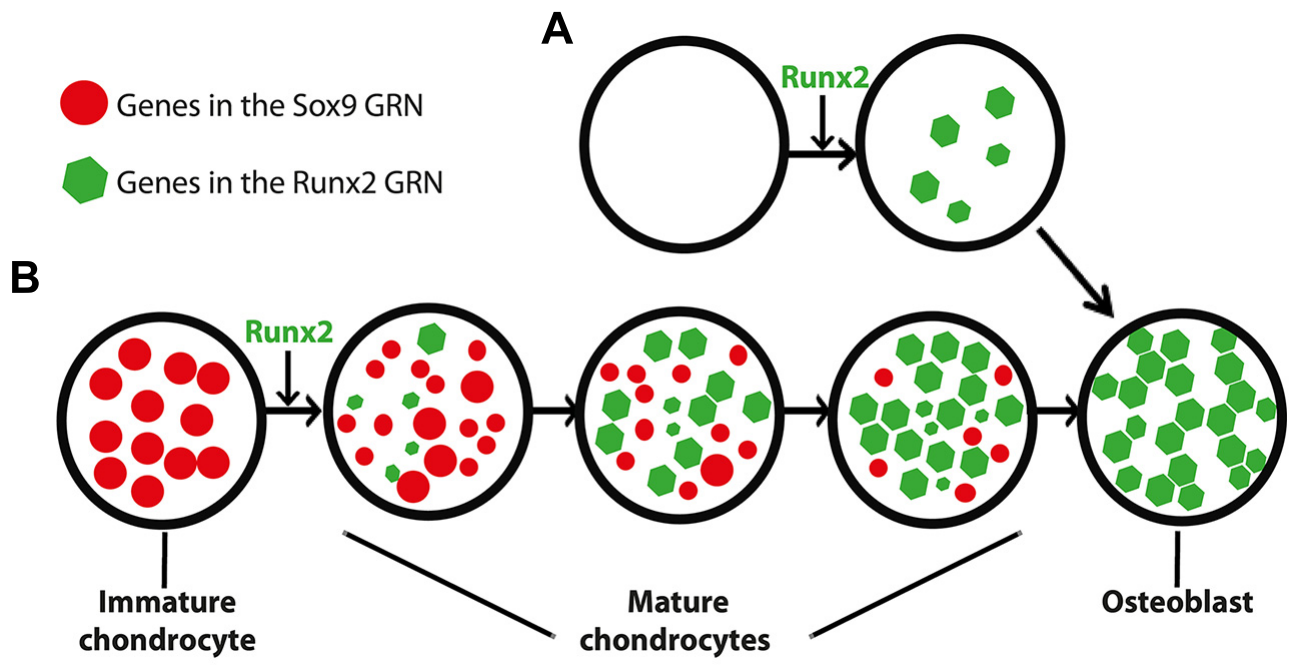
**Differing models for the appearance of the GRN driving osteoblast formation. (A)** In this scenario, the osteoblast (and the Runx2 GRN that drives its formation) appeared *de novo*, independent of the chondrocyte. This model is consistent with saltational evolution, in which large-scale genomic changes may facilitate the evolution of novelty over short periods of geologic time. **(B)** In an alternative scenario, the osteoblast appeared after a series of step-wise additions to the mature chondrocyte (and thus the Runx2 GRN that drives its formation). After establishment of the Runx2 GRN in mature chondrocytes, the osteoblast appeared when another population of cells co-opted the Runx2 GRN. This model is consistent with gradual evolution, in which a series of small changes over geologic time may facilitate the evolution of novelty. The size of the circles and polygons represent relative levels of up- or down-regulation of genes in the respective GRNs (see text for discussion of interactions between Sox9 and Runx2 GRNs).

Combining evidence from traditional and modern studies, we hypothesize that the GRN underlying bone formation evolved from a GRN underlying mature cartilage formation (**Figure 4**). Functional, histological, embryological, and molecular similarities among immature cartilage, mature cartilage, and bone suggest that these tissues may share an evolutionary history (**Figure 1**). The fossil record, comparative anatomy, and embryology demonstrate that immature cartilage evolved first (**Figure 2**). When combined with molecular genetic data (**Figure 3**), this means that the first evolved skeletal GRN was dominated by the gene ancestral to Sox9, driving immature cartilage formation. This GRN likely involved genes ancestral to Runx2 in early phylogenetic (and ontogenetic) stages. In gnathostomes, a Runx2 GRN drives formation of both mature cartilage and bone (**Figure 3**), but how did this novel GRN evolve to produce these novel skeletal tissues?

We propose that immature cartilage provided a structural and molecular “buffer” for the gradual development of this novel, Runx2 GRN. The structural buffering effect refers to the fact that immature cartilage already had a functional role as a skeletal tissue, allowing more freedom for the evolving Runx2 GRN to develop new functions that simply modify a pre-existing skeletal tissue in a gradual, step-wise fashion. The molecular buffering effect refers to the partial dominance of the Sox9 GRN, which might have shielded to some extent the evolving Runx2 GRN from natural selection. This concept recalls the principle of “weak linkage,” which contributes to evolvability by reducing the cost of generating variation (Kirschner and Gerhart, 1998; Gerhart and Kirschner, 2007).

We argue that these putative buffering effects provide a more parsimonious account for the gradual evolution of bone from mature cartilage than the alternative, which depends upon *de novo* establishment of bone in a more saltationist fashion (**Figure 4**). If bone had evolved before mature cartilage, then the Runx2 GRN would have been under much stronger natural selection than if it had been buffered by immature cartilage. Arguments that bone evolved from dentine suffer from the same limitations: how did dentine and its GRN appear? A new GRN appearing simultaneously with a completely new skeletal tissue, while possible, seems a less likely evolutionary scenario than the gradual establishment of the Runx2 GRN during evolution of mature cartilage. Assembling a GRN driving bone formation *de novo* appears to depend upon saltationist genetic mechanisms, such as large-scale genomic changes or small genetic effects acting early in development. Regarding the latter possibility, chondrocytes and osteoblasts are known to share a relatively late embryonic progenitor (Day et al., 2005). Therefore, the former, “macromutational” saltationist mechanism, favored by Goldschmidt (Goldschmidt, 1940), would have to have operated in the *de novo* appearance of the osteoblast. Even saltationists granted that gradualism is the more common evolutionary mechanism (Gould, 2002). Therefore, based on the relative parsimony and abundance of gradualism versus saltationism, we favor a model in which the Runx2 GRN evolved within immature cartilage to produce mature cartilage, and then a different mesenchymal (non-chondrogenic) cell population co-opted this GRN, producing the world’s first example of bone formation (**Figure 4B**).

The hypothesis that bone evolved from mature cartilage also is consistent with a variety of other observations on skeletal tissues (Fisher and Franz-Odendaal, 2012). During evolution, the features of mature cartilage seen in various vertebrate taxa did not appear at the same time (Hall, 1975; Smith and Hall, 1990). Hypertrophy and mineralization occurred first, followed by cartilage matrix degradation, replacement by fat and endochondral bone deposition, and finally, invasion by the vasculature (in tetrapods). These findings suggest that cartilage maturation is a highly evolvable process. Also, the progression from immature cartilage to mature cartilage to bone during evolution is mimicked during endochondral ossification. Recently, cell lineage analyses suggest that some cells that express immature cartilage genes go on to express mature cartilage genes, and finally they express bone genes, effectively transitioning from an immature chondrocyte to a mature chondrocyte to an osteoblast (Hammond and Schulte-Merker, 2009; Zhou et al., 2014; Park et al., 2015). Finally, gene expression patterns appear to overlap much more when comparing mature cartilage to bone in actinopterygians, such as teleosts, than in sarcopterygians, such as tetrapods (Eames et al., 2012). This may reflect differential retention of molecular signatures of the evolutionary history between mature cartilage and bone in earlier diverging versus later diverging vertebrates.

## Comparative Transcriptomics: A Novel Approach to Solve Evo-Devo Issues

Identification of homologous tissue types among different taxonomic lineages using histology and cell morphology has enabled evolutionary studies of histogenesis, but modern molecular techniques will expand dramatically this field. Traditionally, comparative anatomy established homologies at the levels of organs, tissues, and cells. Homology among cartilage-like tissues can be relatively clear for closely related species, but can prove more difficult when comparing distant clades, where clade-specific differences can obscure homology. For example, histological features, such as cellularity of a tissue, may confuse homology designation; cartilage is cellular in vertebrates, but is acellular in hemichordates (Smith et al., 2003; Cole and Hall, 2004; Rychel et al., 2006). In addition, three types of agnathan cartilage have been distinguished by histology: hard cartilage, soft cartilage, and mucocartilage (Zhang and Cohn, 2006; Zhang et al., 2009; Cattell et al., 2011). Which of these would be homologous to hyaline cartilage of gnathostomes, or are they all? Modern evolutionary thinking overlooks such superficial histological differences, emphasizing instead the importance of tracking changes to the underlying molecular genetic factors during trait evolution.

Evolutionary studies of skeletal cells will benefit from transcriptomic techniques, such as RNAseq, that enable characterization of their molecular fingerprints, which are the sets of genes expressed in a homogenous population of cells (Arendt, 2003). Comparing the molecular fingerprint of distinct cell types has yielded insight into evolutionary relationships among remote animal clades (Arendt, 2005, 2008; Eames et al., 2012). A few technologies can generate molecular fingerprints, but of these, RNAseq currently produces the most robust, unbiased results (Necsulea and Kaessmann, 2014). Some advantages of RNA-seq include a higher dynamic range, allowing the detection of transcripts that are expressed at very high or low levels, and the ability to detect novel genes and alternative splice variants in samples from any animal (Wang et al., 2009). Important for evolutionary studies, then, RNAseq allows for an accurate comparison of molecular fingerprints in both closely and distantly related species (Necsulea and Kaessmann, 2014; Pantalacci and Semon, 2015).

Tracking gene expression patterns that underlie a homologous trait through phylogeny provides unparalleled insight into molecular mechanisms of evolution. In fact, comparative transcriptomics might reveal that two tissues are homologous (so-called “deep homology”; Shubin et al., 2009), despite superficial histological or cellular differences. For example, the presence of immature cartilage in a variety of invertebrate taxa raises the possibility of a tissue with deep homology to cartilage present in the ancestor to all metazoans (**Figure 2B**). Also, identifying invertebrate tissues that express “bone genes” may reveal deep homology of these cells to osteoblasts, potentially facilitating the *de novo* appearance of the Runx2 GRN underlying bone formation. Genes in the vertebrate *Sparc* family play a role in skeletal matrix mineralization *in vitro* (Termine et al., 1981; Pataquiva-Mateus et al., 2012). Although similar *in vivo* roles for *Sparc* genes have not been demonstrated clearly (Roach, 1994; Gilmour et al., 1998; Rotllant et al., 2008), comparative genomics reveal a clear correlation between some *Sparc* genes and bone formation (Kawasaki and Weiss, 2006; Martinek et al., 2007; Koehler et al., 2009; Bertrand et al., 2013; Venkatesh et al., 2014). Interestingly, *Sparc* genes are expressed in amphioxus, which do not have bone nor mineralize their tissues (Bertrand et al., 2013). If Runx2 co-opted regulation of these genes during the *de novo* appearance of the osteoblast, then *Sparc*-expressing cells in amphioxus may have deep homology to osteoblasts.

Comparative transcriptomics can be used to evaluate quantitatively important features of GRN evolution, including constraint and adaptation. Although Gould recently revived the formalist pleas of Galton, Whitman, and others for constraint to have a positive role during evolution (Gould, 2002), constraint commonly is considered a restriction or limitation on the evolutionary process (Arnold, 1992). Evidence of constraint can be seen when transcriptomes are highly conserved among various tissues or clades, presumably due to genomic, developmental, or structural limitations. In addition to these constraints, a GRN under stabilizing selection would not vary much with respect to the genes expressed and their levels of expression, thus giving a transcriptomic signal of constraint. In fact, the architecture of GRN kernels, which usually consist of transcription factors and other regulatory genes, can remain highly conserved for a long period of time (Levine and Davidson, 2005; Davidson and Erwin, 2006). In contrast, adaptation commonly is considered positive for change during evolution (Gould, 2002; Stayton, 2008; Losos, 2011). Evidence of adaptation can be seen when transcriptomes differ widely among various tissues or clades, presumably in response to tissue- or clade-specific selective pressures. A GRN under negative or positive selection would vary a lot in the genes expressed and their levels of expression.

Comparative transcriptomics has unraveled the complexity of several important developmental and evolutionary processes in both invertebrate (Levin et al., 2012; McKenzie et al., 2014) and vertebrate organisms (Chan et al., 2009; Brawand et al., 2011). A major challenge in evolutionary biology is to explain the appearance of novel traits and the GRNs underlying their formation. Two different models have been proposed, with only one currently receiving much experimental support. In the first model, a GRN driving a novel trait also evolved *de novo* (**Figure 4A**). For example, orphan genes, or genes without clear family members, might be important drivers of evolutionary novelty. First described in the yeast genome (Dujon, 1996), they occur also in many taxa, including rodents, primates, and humans (Heinen et al., 2009; Toll-Riera et al., 2009a,b; Li et al., 2010). Orphan genes might have appeared *de novo* from non-coding sequences rather than from existing genes (Tautz and Domazet-Loso, 2011). Subsequent interactions that these orphan genes establish among other genes would create a novel GRN with the capability of driving formation of a novel trait. This “*de novo*” model has received little experimental support in metazoans, but currently serves as the basis for the hypothesis that bone (or dentine, if dentine appeared before bone during evolution) evolved before mature cartilage (**Figure 4A**). In molecular terms, the GRN driving formation of the osteoblast would have appeared *de novo*, presumably in a short evolutionary timeframe.

In the second model for appearance of evolutionary novelties, which is increasingly supported by the literature, a novel trait appears by co-opting a pre-existing GRN (**Figure 4B**; Fisher and Franz-Odendaal, 2012; Achim and Arendt, 2014). For example, comparative genomic studies on muscle cells, immune cells, and neurons suggested that these cell types evolved by co-opting pre-existing genetic systems (Achim and Arendt, 2014). In addition, the appearance of a novel embryonic cell lineage in vertebrates, the neural crest cell, has been argued to result from the co-option of pre-existing GRNs that were employed by cells in the neural tube, notochord, and pharynx in ancestral chordates (Baker and Bronner-Fraser, 1997; Donoghue and Sansom, 2002; Meulemans and Bronner-Fraser, 2005, 2007; McCauley and Bronner-Fraser, 2006; Zhang and Cohn, 2006). In fact, the neural crest-derived vertebrate cartilaginous head skeleton might have arisen after neural crest cells co-opted an ancestral chordate GRN that was used for cartilage formation in other parts of the body (Jandzik et al., 2015). Here, we use the same argument to support our idea that the osteoblast appeared when a non-chondrogenic mesenchymal cell co-opted expression of the mature cartilage Runx2 GRN.

## Comparative Transcriptomics and Skeletal Tissue Evolution

How extensive is our understanding of the GRNs driving cartilage and bone formation? As outlined above, Sox9 and Runx2 GRNs are critical in a variety of vertebrates, but is this the whole story? Few studies have analyzed the molecular fingerprint of the chondrocyte and osteoblast using unbiased transcriptomics, but such experiments may identify unknown GRN’s driving formation of these cell types. The chondrocyte molecular fingerprint was estimated by compiling data from the literature and summarizing their interactions into a GRN (Cole, 2011). Recently, transcriptomics on Sox9 and Runx2 loss-of-function skeletal cells *in vitro* have shed light on Sox9 and Runx2 GRNs that are relevant to chondrocyte and osteoblast differentiation (Oh et al., 2014; Wu et al., 2014). A promising future direction is to use transcriptomics to define these GRNs *in vivo* using *Sox9* and *Runx2* loss-of-function animals. Comparative transcriptomics between vertebrae and gill arch skeletal elements of a teleost demonstrated a high degree of overlap in gene expression between these two tissues (Vieira et al., 2013), but the presence of multiple cell types, including chondrocytes and osteoblasts, in both samples confounds attribution of these data to a particular cell type. Therefore, more specific techniques should be used to isolate a pure population of cells *in vivo* in order to accurately reveal and compare the molecular fingerprints of different skeletal cell types (**Figure 3**).

**FIGURE 5:**
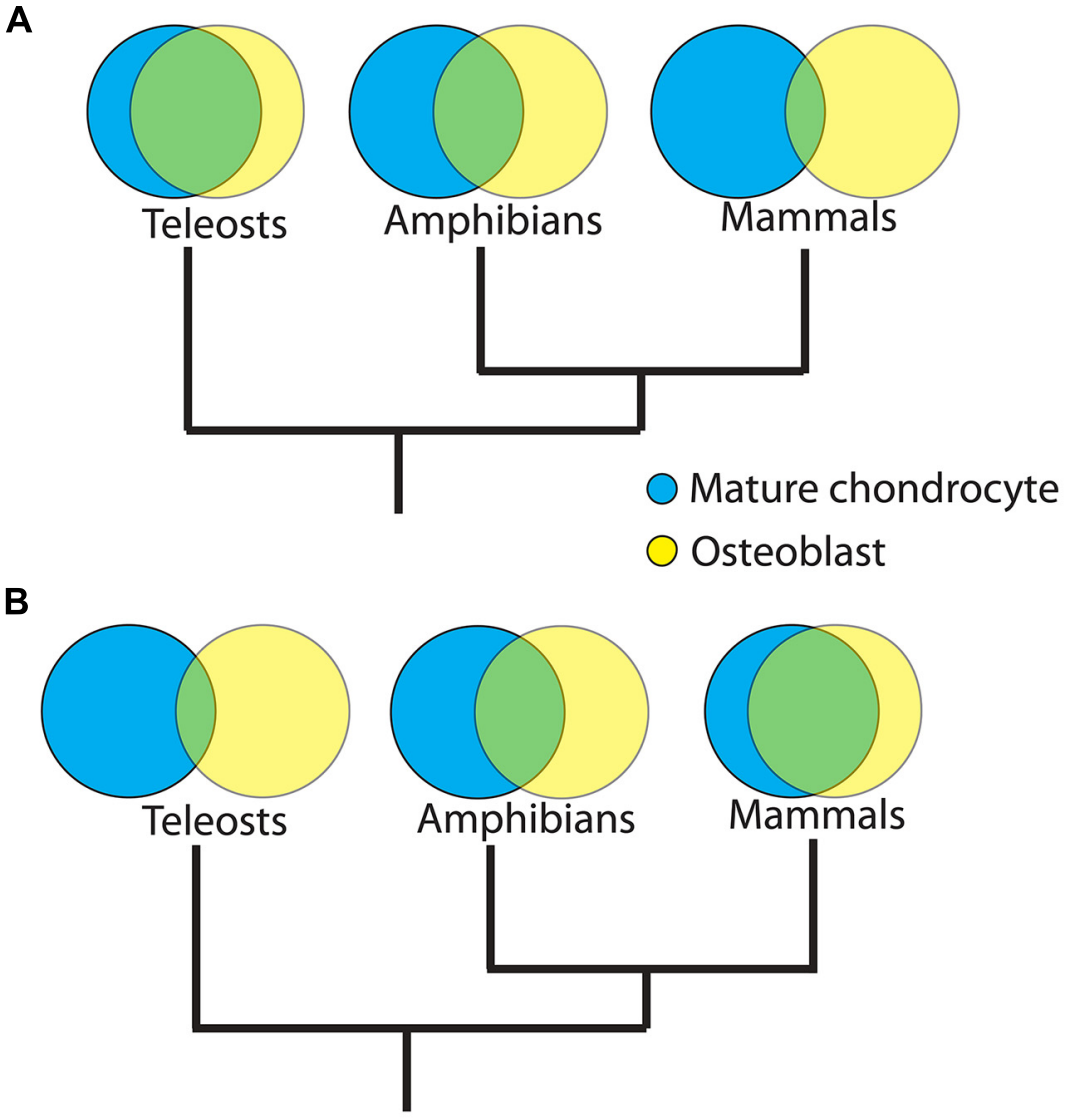
**Divergent vs. convergent evolution of the molecular fingerprints of mature chondrocytes and osteoblasts.** Venn Diagrams comparing putative molecular fingerprints between mature chondrocytes and osteoblasts in three distinct vertebrate clades may resolve among two hypotheses for the origins of the osteoblast. **(A)** Divergent model. Osteoblast evolved when a GRN was co-opted from mature chondrocytes. Differing selective pressures on ancestors of various lineages, followed by lineage-specific constraints, may have caused gradual divergence between the GRN of osteoblasts and mature chondrocytes during vertebrate evolution. If true, then the overlap between mature chondrocyte and osteoblast molecular fingerprints will be significantly higher in earlier diverged lineages, such as teleosts, than in later diverged lineages, such as mammals. **(B)** Convergent model. Osteoblast GRN evolved *de novo*. Similar selective pressures on osteoblasts and mature chondrocytes in ancestors of later diverging lineages may have caused convergence between the GRN of osteoblasts and mature chondrocytes during vertebrate evolution. If true, then the overlap between molecular fingerprints of mature chondrocytes and osteoblasts will be significantly lower in earlier evolved lineages. Branch lengths in trees are arbitrary; the overlap between molecular fingerprints is shown in green and, in the divergent model, may represent the ancestral GRN kernel of both mature chondrocyte and osteoblast.

Two related, fascinating questions remain for future research: how did the GRNs directing skeletal cell differentiation appear, and how did they evolve afterward? In this review, we argue that gradual establishment of the Runx2 GRN during evolution of the mature chondrocyte (subsequently co-opted by a non-chondrogenic mesenchymal cell to form bone) is more parsimonious than the *de novo* appearance of the Runx2 GRN in osteoblasts (**Figure 4**). Given the latter possibility, however, the tremendous gene expression similarities between mature cartilage and bone in tetrapods also may reflect co-option of the Runx2 GRN by the mature chondrocyte after it was established in the osteoblast. These possibilities predict divergent vs. convergent evolution, respectively, of the Runx2 GRN in mature chondrocytes after the appearance of the osteoblast. Therefore, we propose an examination of skeletal cell molecular fingerprints in a variety of vertebrates to resolve this issue. Our divergent model predicts that the overlap between mature chondrocyte and osteoblast molecular fingerprints will decrease in more recently evolved organisms (**Figure 5A**). For example, molecular fingerprints of mature chondrocytes and osteoblasts would overlap more in earlier diverged lineages of vertebrates, such as teleosts, than in later evolved lineages, such as amphibians or mammals. On the other hand, the convergent model predicts the opposite result (**Figure 5B**).

**FIGURE 6:**
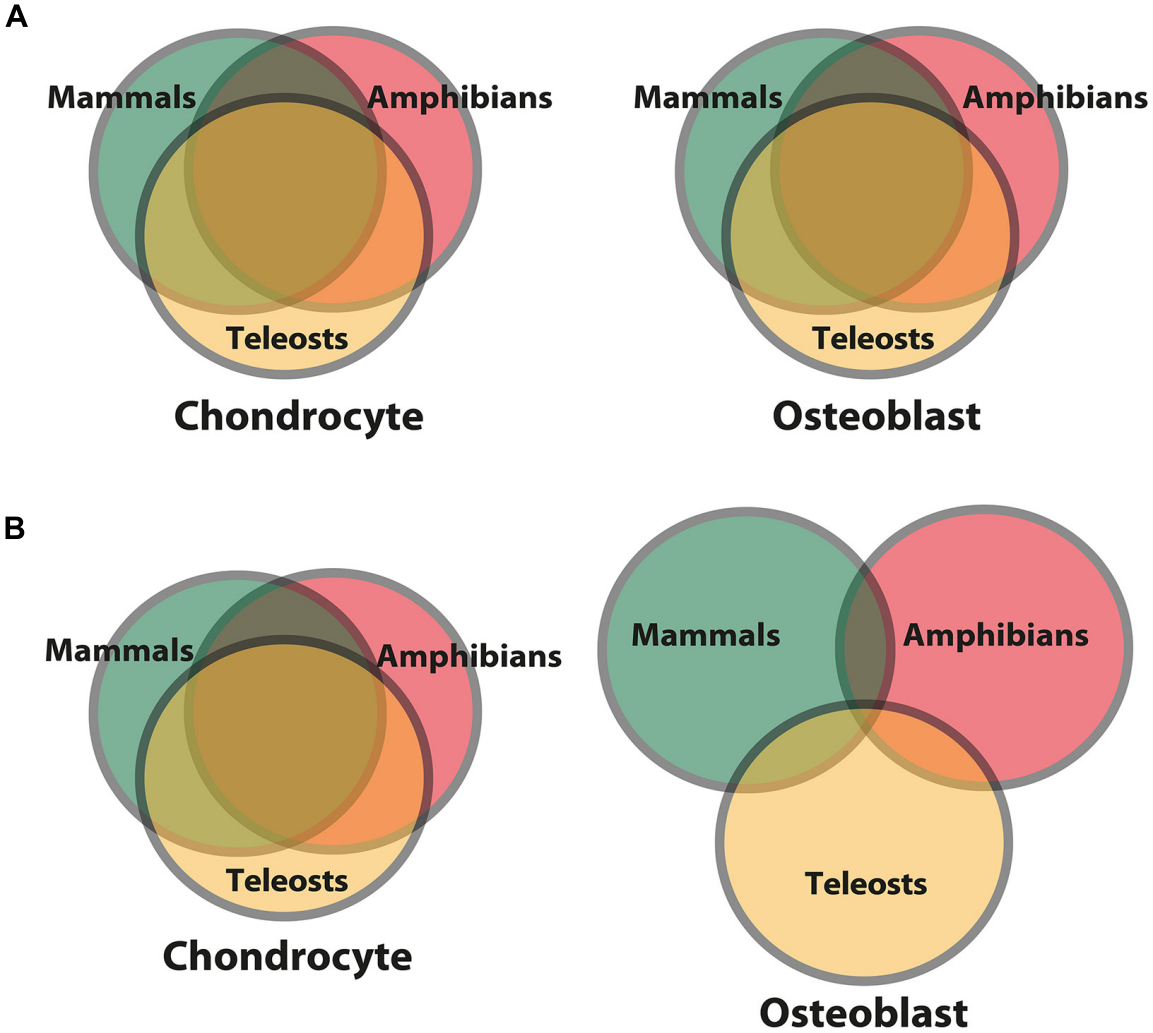
**Differing models for levels of constraint and adaptation among skeletal cells of different vertebrate lineages.** Venn diagrams comparing putative molecular fingerprints of chondrocytes and osteoblasts from three vertebrate clades. The ancestral chondrocyte and osteoblast GRN kernels are represented in the overlap of the circles. More overlap represents more constraint/less adaptation among clades. **(A)** The first scenario predicts that the molecular fingerprints of the chondrocyte and osteoblast (and thus the GRNs governing their formation) are constrained to equal extents among vertebrates (Fisher and Franz-Odendaal, 2012; Vieira et al., 2013). **(B)** The second scenario predicts that the chondrocyte molecular fingerprint is more constrained among vertebrate clades, while the osteoblast molecular fingerprint shows more signs of clade-specific adaptations (Eames et al., 2012). In general, this latter scenario posits that a cell type appearing later during animal phylogeny is more free to vary than a cell type appearing earlier, whose molecular fingerprint was fixed via stabilizing selection.

But do skeletal cell molecular fingerprints evolve in clade-specific manners? A limited number of studies trying to answer this question suggest two competing ideas. On the one hand, molecular fingerprints of the chondrocyte and the osteoblast have been proposed to be highly constrained among various vertebrate clades (**Figure 6A**; Fisher and Franz-Odendaal, 2012; Vieira et al., 2013). On the other hand, gene expression comparisons between gar, zebrafish, chick, and mouse suggest that the chondrocyte molecular fingerprint is constrained among vertebrates, while the osteoblast molecular fingerprint varied, perhaps in response to clade-specific selective pressures (**Figure 6B**; Eames et al., 2012). Interestingly, generalizing these results puts forward the hypothesis that earlier-evolved cell types, in this case chondrocytes, might be more constrained in their gene expression than cell types that appeared later, such as osteoblasts, perhaps due to stabilizing selection over geologic timescales. Comparative transcriptomics can quantitate constraint and adaptation, by measuring how transcript levels vary among samples from different taxonomic lineages.

In the future, comparative transcriptomics will elucidate the dynamics of skeletal cell type evolution, identifying lineage-specific changes in gene expression, providing quantitative measures of constraint and adaptation, and potentially establishing deep homology of skeletal cells with previously unappreciated cell types. Indeed, appropriate application of comparative transcriptomics has the potential to revolutionize understanding of the molecular mechanisms of trait evolution.

## Summary

Given the role that fossilized bones played in devising early evolutionary theory, skeletal tissue evolution has fascinated scientists for centuries. In particular, the appearance of bone as an evolutionary novelty demands explanation, which modern molecular and embryological techniques address in ways never imagined by studies of the fossil record alone. Here, we focus on the three main skeletal tissues present in vertebrates (immature cartilage, mature cartilage, and bone), and use findings from both traditional and modern studies to argue that bone evolved from mature cartilage. Standing in contrast to the available fossil record, which suggests that bone appeared prior to mature cartilage, this hypothesis posits that a GRN driving traits such as matrix mineralization in mature cartilage was co-opted by non-chondrogenic mesenchymal cells to produce bone. Alternatively, the GRN driving bone formation may have evolved first and subsequently was co-opted by mature cartilage, but we use an argument based on parsimony that this scenario would be more complicated to achieve. Comparing the molecular fingerprints of skeletal tissues in agnathans and sister chordate species with those in vertebrates might resolve among these possibilities. In addition to comparative transcriptomics revealing the origins of evolutionary novelties, tracking molecular fingerprints of skeletal cells in various vertebrate lineages can identify quantitative measures of constraint and adaptation within the GRNs that govern the formation of skeletal tissues. Therefore, we strongly believe that this novel approach may revolutionize understanding of the evolution of cartilage and bone and more generally provide a modern paradigm for molecular genetic changes during the evolutionary process.

## Conflict of Interest Statement

The authors declare that the research was conducted in the absence of any commercial or financial relationships that could be construed as a potential conflict of interest.
